# Plasma oxidized LDL and thiol-containing molecules in patients with exudative age-related macular degeneration

**Published:** 2010-12-06

**Authors:** Alireza Javadzadeh, Amir Ghorbanihaghjo, Elham Bahreini, Nadereh Rashtchizadeh, Hassan Argani, Samira Alizadeh

**Affiliations:** 1 Biotechnology Research center, Tabriz, Iran; 2 Drug Applied Research Center, Tabriz University of Medical Sciences, Tabriz, Iran

## Abstract

**Purpose:**

It was proposed that total thiols (tSH) as powerful reducing agents and oxidized low-density lipoprotein (OX-LDL) may be associated with development of choroidal neovascularization in exudative age-related macular degeneration (E-ARMD).

**Methods:**

In a case-control study, 45 patients with E-ARMD were compared with 45 sex- and age-matched healthy controls. The levels of plasma homocysteine (Hcy) and OX-LDL as oxidant agents, and of tSH and glutathione (GSH) as antioxidant markers, were estimated in E-ARMD patients and controls.

**Results:**

The levels of Hcy (15.4±7.2 μM versus 10.7±3.7 μM; p=0.001) and OX-LDL (52.2±13.8 U/l versus 37.8±10.8 U/l; p=0.001) were statistically higher, while GSH (1.10±0.97 μM versus 2.09±1.04 μM; p=0.001) and tSH (0.31±0.06 mM versus 0.35±0.05 mM; p=0.001) were statistically lower, in the patients with E-ARMD than in the control group, respectively. The plasma OX-LDL concentration also exhibited a positive and significant correlation with Hcy (r=0.719, p=0.001) in patients with E-ARMD.

**Conclusions:**

Lower GSH and tSH as antioxidant and higher Hcy levels as oxidant agents in E-ARMD patients may have resulted in an oxidative environment that was associated with OX-LDL. Further studies with more cases are required to confirm the hypothesis.

## Introduction

Age-related macular degeneration (ARMD) is one of the leading causes of blindness among the elderly throughout the world [1]. It is a complex, multifactorial disease that affects the central region of the retina. The prevalence of early ARMD increases from around 4% in those younger than 60 years to more than 30% in those 85 years and older [2]. There are two forms of ARMD, nonexudative (dry) and exudative (wet). The nonexudative form is more common, and accounts for most ARMD cases. The exudative form (E-ARMD), however, is more debilitating, and is responsible for more than 80% of the visual loss in such patients. New choroidal vessel formation, vascular leakage, and hemorrhage are the hallmarks of E-ARMD [3]. It is also associated with degenerative, oxidative, and inflammatory changes in the macular region of the retina. Genetic factors, oxidative stress, ischemia, aging of the retinal pigment epithelium, and inflammation are believed to be the main etiological factors of E-ARMD [4]. In recent genetic investigations, Hageman et al. reported that a variation in the factor H gene increases the likelihood of developing ARMD [5]. There are genetic predisposing factors, but these do not fully account for the mechanism of the disease. These genetic factors may modify the severity of the ARMD phenotype. Oxidative stress is among the modifiers that can lead to changes in the structure of the macular region [6].

There is good evidence that oxidative stress is involved in the pathogenesis of ARMD [7]. Oxidative stress results in the generation of free radicals, leading to the promotion of lipid peroxidation. This affects not only low density lipoprotein (LDL) and other lipoproteins, but also cellular lipids, including those in arterial walls and macrophages [8]. Increases in oxidative products contribute to atherosclerosis and vasomotor tone. Vine et al. showed that elevated CRP and homocysteine levels are associated with ARMD and that they contribute to inflammation and atherosclerosis in the pathogenesis of ARMD [9]. Essentially, retinal hypoxia and cumulative oxidative stress contribute to aging processes (e.g., reduced ability for cell division and altered enzyme activity). The hypoxia of retinal cells then induces the formation of the vascular endothelial growth factor. Vascular and oxidative stress theories have been implicated for the development of choroidal neovascularization in E-ARMD [10,11].

Thiols are powerful reducing agents that are capable of acting as antioxidants in vivo. Thiols exist in three forms: free-thiol and two types of disulfides, namely, homodisulfides and heterodisulfides. Several aminothiols, e.g., cysteine, homocysteine (Hcy), and glutathione (GSH), and disulfides (e.g., cystine, homocystine, and oxidized glutathione), interact by means of redox and disulfide exchange [12]. This dynamic system (with respect to thiol status) is important for normal physiologic function [13]. Changes in the redox thiol status lead to the induction of oxidative stress and apoptosis. As both an intracellular and extracellular redox buffer, tSH plays important roles in the in vivo prevention of atherosclerosis [14].

Hcy, an amino acid that is found in the blood, is an independent and potentially modifiable risk factor for various forms of vascular disease. Epidemiological studies have reported that the level of Hcy in blood is related to higher risk of coronary heart disease, stroke, and peripheral vascular disease. Other evidence suggests that Hcy may have an effect on atherosclerosis by damaging the inner lining of arteries and promoting blood clots [15,16]. The Hcy concentration in the blood of healthy individuals varies with age, geographical area, and genetic factors; its concentration normally increases with age [17].

The adverse effects of Hcy on endothelial function may be mediated by (1) the reduced production and bioavailability of nitric oxide as a result of oxidant stress [18], with the formation of reactive oxygen species [19], including superoxide anion and hydrogen peroxide, increased lipid peroxidation [20], (2) impaired production of the antioxidant glutathione peroxidase [21] and (3) more important by its metabolite, homocysteinethiolacton. Coral et al. [22]. showed that Hcy is a well known inducer of the vascular endothelial cell damage associated with extracellular matrix changes and increased collagen turnover

The cellular uptake of oxidized low-density lipoprotein (OX-LDL) by macrophages and vascular endothelial cells plays a crucial role in the pathogenesis of atherosclerosis [21]. Because the pathologic changes in E-ARMD are similar to those in atherosclerosis [23,24], and because atherosclerosis may contribute to the pathogenesis of E-ARMD [25,26], we hypothesized that oxidized lipoproteins in the macular area of eyes with E-ARMD may be improved, just as is intima in cases with atherosclerosis that is detectable by OX-LDL levels in blood.

Because a relationship exists between hyperhomocysteinemia and atherosclerotic disease, we designed this study to evaluate the total levels in plasma of Hcy and OX-LDL acting as oxidant agents, as well as levels of other related physiologic thiol compounds acting as antioxidant markers in E-ARMD.

## Methods

### Study population

This case-control study was conducted in the Retina clinic (Department of Ophthalmology, Tabriz University of Medical Sciences), between June 2008 and January 2009. The study groups were composed of 90 individuals ranging in age between 50 and 80 years, enrolling 45 patients with E-ARMD (18 men and 27 women; mean age: 71±7 years) and 45 healthy matched controls (18 men and 27 women; mean age: 69±5 years). Data regarding age and sex, and a detailed medical history, were recorded. All participants underwent a complete ophthalmic examination consisting of the best corrected visual acuity (using Snellen-Chart and calculated as the logarithm of the minimal angle of resolution, notated “logMAR”), slit-lamp biomicroscopy, dilated funduscopy (using a Slit-lamp, Haag-Streit, R 900; Haag-Streit AG, Switzerland with a super-field indirect lens), fundus photography, and fundus fluorescein angiography (Imagenet 2000; Topcon TRC 50IX; Topcon Corp., Japan) when necessary. Recruited patients in the study had either a classic form of choroidal neovascularization or a disciform scar. To limit the effects of interference factors in our results, we excluded other pathological ophthalmic conditions, such as trauma and angle-closure glaucoma. We also excluded those who were in an oxidative condition, such as smokers, subjects who were on antioxidant supplements, and subjects with systemic disorders such as diabetes, any inflammatory processes, and renal and/or liver dysfunction.

The ethics committee at the Tabriz University of Medical Sciences reviewed and approved the present study, in compliance with the Declaration of Helsinki. Informed consent was obtained from all participants.

### Sampling and primary tests

Participants’ venous blood samples were obtained the morning following overnight fasting. The sera or plasma were separated immediately and then analyzed using enzymatic assays with an automated chemical analyzer (Abbott Analyzer; Abbott Laboratories, Abbot Park, IL) for serum glucose, urea, creatinine, alanine aminotransferase, aspartate aminotransferase. Serum lipid profiles, including total cholesterol, triglycerides, and high-density lipoprotein (HDL) cholesterol. Low-density lipoprotein (LDL) cholesterol was calculated using the Friedewald equation [27]. The remaining samples were stored at −70 °C pending analysis.

### Homocysteine

Plasma Hcy concentrations were determined using a commercially available enzyme-linked immunoassay (Axis-Shield, Axis Biochemical ASA, Distributed by IBL, Hamburg, Germany). With this method, total free and protein-bound circulating Hcy moieties are reduced to free Hcy with dithiothreitol. The Hcy was then converted to *S*-adenosyl-L-homocysteine (SAH) by using SAH hydrolase and excess adenosine. After the addition of an anti-SAH antibody, in the next stage, a secondary rabbit anti-mouse antibody was labeled with the enzyme horseradish peroxidase. The peroxidase activity was measured spectrophotometrically after the addition of the substrate. The absorbance was inversely related to the concentration of Hcy in the sample.

### Total thiol levels in plasma

Plasma tSH levels were determined using a spectrophotometric method, as previously described by Hu [28].

### Glutathione

Plasma GSH levels were measured using an Enzyme immune assay (EIA) method (Cayman Chemical Company, Ann Arbor, MI).

### Oxidized low density lipoprotein

Plasma OX-LDL levels were measured using an enzyme-linked immunosorbent assay (ELISA) kit (Mercodia, Uppsala, Sweden).

### Statistical analysis

Statistical analyses were performed using statistical package for the social sciences (SPSS) for Windows (Version 16, SPSS, Inc., Chicago, IL). The Kolmogorov–Smirnov test was used to evaluate the distributions, and results were expressed as means±standard deviation. The independent *t*-test was used to assess the significance of the differences between the two groups. The correlations between OX-LDL and Hcy were evaluated using the Pearson test; p<0.05 was considered statistically significant.

## Results

**Figure 1:**
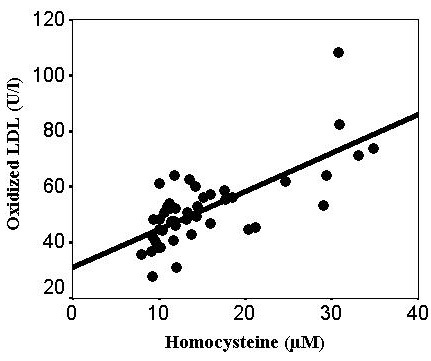
Plasma concentrations of oxidized low-density lipoprotein (oxidized LDL) versus homocysteine levels in the patient subjects. There was a direct linear correlation between the oxidized LDL and homocysteine levels in the patient group (r=0.719, p=0.001).

**Figure 2:**
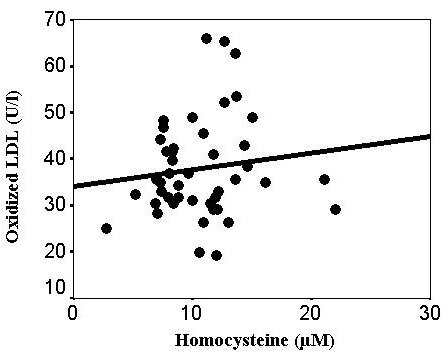
Plasma concentrations of oxidized low-density lipoprotein (oxidized LDL) versus homocysteine levels in control subjects (r=0.123, p=0.422).

The table summarizes the clinical characteristics and laboratory findings from the patients with E-ARMD and from the controls. Both groups were matched with regard to age and gender. As shown in the Table 1, the total plasma Hcy concentration was significantly higher (71%) among the patients with E-ARMD than among the controls (15.4±7.2 μM versus 10.7±3.7 μM; p=0.001). The plasma levels of GSH were significantly lower: almost 50% lower in the patients with E-ARMD (1.10±0.97 μM) than in the controls (2.09±1.04 μM; p=0.001). The differences between the plasma tSH levels for patients with E-ARMD and the controls were significant (0.31±0.06 mM versus 0.35±0.05 mM; p=0.001, respectively). The total plasma OX-LDL level in the patients with E-ARMD was 40.5% higher than the controls (52.2 ± 13.8 U/l versus 37.8±10.8 U/l; p=0.001). The plasma OX-LDL concentration had a significant positive correlation with Hcy (r=0.719, p=0.001, Figure 1) in the patients with E-ARMD, but not in the controls (r=0.123, p=0.422, Figure 2).

**Table 1:** Demographic data for the E-ARMD patients and controls

**Parameters**	**Patients (mean±SD)**	**Controls (mean±SD)**	p value*
Number /Sex	45/(18M, 27 F)	45/(18M, 27 F)	-
Age (year)	71±7	69±5	0.11
BCVA^a^	1.18±0.36	0.10±0.07	0.001
Pseudophakic	34	29	-
BMI^b^ (Kg/m²)	25.3±3.8	27.3±5.6	0.11
Systolic pressure (mmHg)	129.8±20	124.4±7.2	0.12
Diastolic pressure (mmHg)	77.5±10	82.5±8.1	0.11
Glucose (mg/dl)	88.0±13	85.2±14.9	0.58
Urea (mg/dl)	32.3±7.2	34.3± 6.1	0.16
Creatinine (mg/dl)	1.0±0.2	1.08±0.18	0.14
Triglycerides (mg/dl)	144.5±63.8	153.7±49.5	0.45
Cholesterol (mg/dl)	204.0±39.5	181.1±36.3	0.005
HDL-C^c^ (mg/dl)	43.1±5.0	44.2±6.3	0.34
LDL-C^d^ (mg/dl)	131.6±40.2	105.1±35.3	0.001
Homocystein (μM)	15.4±7.2	10.7±3.7	0.001
Gluthation (μM)	1.10±0.97	2.09±1.04	0.001
Total thiol (mM)	0.31±0.06	0.35±0.05	0.001
OX-LDL^e^ (U/l)	52.2±13.8	37.8 ±10.8	0.001

^a^BCVA = best corrected visual acuity, ^b^BMI = body mass index, ^c^HDL-C = high-density lipoprotein cholesterol, ^d^LDL-C = low-density lipoprotein cholesterol, ^e^OX-LDL = oxidized LDL *Independent-sample T test.

## Discussion

Macula is particularly susceptible to oxidative stress because of the high number of poly unsaturated fatty acids (PUFAs) in its cell membranes, its exposure to direct light, and its high consumption of oxygen [29]. As a result, the macula is highly susceptible to ROS. Formation of ROS could lead to lipid peroxidation, which initiates an inflammatory response [30]. Consequently, increased plasma OX-LDL as an oxidant agent may suggest the presence or risk of ARMD more precisely than other serum factors. Oxidative stress disrupts the retinal pigmented epithelial (RPE) cell junctions and the integrity of the blood-retina barrier, both of which contribute to neovascular processes. Thus, oxidative stress should be considered a risk factor for new vessel formation [31,32].

The main findings of this case-control study was that higher levels of Hcy as an oxidant agent and lower GSH and tSH concentrations as antioxidative markers were detected among the patients with E-ARMD, compared with age- and gender-matched healthy controls. These results are confirmed by Coral et al.’s outcomes in a study on exudative ARMD patients. They revealed that there was a significant, elevated Hcy level and diminished thiol pool content in E-ARMD [33]. Many studies have shown that the redox states of human plasma thiol compounds can transform into oxidized states in older age groups [34]. tSH plays a prominent role in antioxidant reactions, and in catalysis, regulation, and electron-transport reactions, and in reactions that preserve the correct structure of proteins. Mixed disulfides with proteins are formed by reaction of S-thiolation, in which protein thiols conjugate with non-protein thiols [35]. This process plays a regulatory and an antioxidant role, since it protects protein −SH groups against irreversible oxidation from −SO_2_H and −SO_3_H; moreover, it participates in signal transduction [36].

Patients in our study had mild hyperhomocysteinemia, a state has been described in a study on the Iranian population by Ghaedi et al. [37]. In that study, the reference intervals for plasma Hcy were determined. The Hcy means in cases and controls were 15.56±6.77 and 11.51±4.63, respectively. The plasma level of Hcy in the 100 normal individuals had a mean of 11.51, and the 95 percentile of cases were in the ranged of 7.8 to 16.1. Cross-sectional and prospective studies have shown that even mildly increased blood Hcy levels may play a role as an oxidative agent, linked with an increased risk of cardiovascular disease [38]. Wotherspoon et al. [39] suggested that hyperhomocysteinaemia might cause vascular damage by increased oxidant stress and mechanisms other than endothelial dysfunction. Impaired endothelial function has been demonstrated in healthy subjects with mild to moderate fasting hyperhomocysteinaemia. Kamburoglu et al. revealed increased plasma Hcy levels in both the exudative and dry subtypes of ARMD, and decreased vitamin B12 levels in exudative ARMD. They also suggested an association between elevated plasma homocysteine and ARMD, regardless of the subtype [40]. In a meta-analysis of 30 studies and more than 6,000 events, a 25% lower Hcy level was associated with an 11% lower risk of ischemic heart disease and a 19% lower risk of stroke [41]. These findings have potentially important clinical implications, because plasma homocysteine levels can be safely lowered by 25% with folic acid, and antioxidants such as vitamins C and E may reduce vascular risk in these high-risk patients [40].

Hcy has been shown to undergo autoxidation and to lead to the generation of reactive oxygen species (ROS), such as the superoxide anion (O_2_^–^) and hydroxyl radical (OH°). These oxidative radicals initiate the oxidation of LDL-C, which induces vascular dysfunction and accelerates atherosclerosis [42]. Homocysteine thiolactone, one of several Hcy metabolites, can also accelerate atherogenesis via homocysteinylated LDL-C (by attaching to apo-B100), which may be more susceptible to oxidation than native lipoproteins [43]. Homocysteinylated LDL-C decreases endothelial Na^+^, K^+^-ATPase activity, leading to an intracellular sodium overload, and a subsequent increase in calcium ion concentrations. A high concentration of intracellular calcium reduces the production of nitric oxide (NO) and increases the generation of peroxynitrite (ONOO^–^), a highly reactive nitrogen species that is derived from NO and from superoxide anion radicals [44,45].

In the present study, the oxidant agent OX-LDL’s levels were higher in the E-ARMD patients than in the healthy controls. We assumed that the increased plasma OX-LDL concentration was attributable to the hyperoxidative state in E-ARMD patients. LDL homocysteinylation causes the overproduction of OX-LDL. Endothelial dysfunction is due to the action of OX-LDL, which subsequently causes atherosclerotic plaque development. Kamei et al. [46] explained that oxidized lipoproteins and macrophages were colocalized in ARMD lesions, and that most macrophages in the choroidal neovascularization membranes expressed oxidized lipoprotein-specific scavenger receptors. Because the uptake of OX-LDL by macrophages via scavenger receptors and the accumulation of lipid-laden foam cells in the arterial intima are key events in early atherogenesis, their findings suggested that macrophages may also accumulate in the area of the ARMD, possibly to phagocytose oxidized lipoproteins through scavenger receptors [21,24]. The relationship between lipid peroxidation and hyperhomocysteinemia suggests that the generation of reactive intermediates by thiol oxidation may be one of the most important mechanisms in this context [14].

Maintaining the intracellular thiols, such as GSH, in their reduced form, may allow for the maintenance of Hcy and other intracellular thiols in redox states [47]. In this study, low plasma levels of GSH and thiol were apparent in E-ARMD patients, compared with healthy individuals. Since the plasma GSH concentration reflects its levels in various tissues, a reduced plasma concentration of GSH may be a diagnostic indicator of a pathological state [48].

GSH is the most important endogenous antioxidant in humans. It is often accompanying by other endogenous thiols, such as cysteine, cysteinylglycine and even Hcy (in low concentration). These thiols scavenge ROS and are involved in preserving the pro-oxidant–antioxidant balance in human tissues [49]. It has been reported that GSH and thiol content may decrease in certain pathological states and on account of the aging process. This causes Hcy oxidation to become uncoupled, which leads to oxidative damage to the endothelial cells [50]. In fact, several studies have demonstrated that a high dietary intake of anti-oxidative agents, such as beta carotene, vitamins C and E, and zinc, may be associated with a substantially reduced risk of E-ARMD in elderly persons [51]. This effect was also found by the Age-Related Eye Disease Study Research Group [52-55].

Although thiols protect cells from oxidative stress by scavenging reactive intermediates, they have ominous side effects that make their clinical application potentially harmful. In the presence of metal ions (such as Fe^++^) and oxygen, they can be autoxidized, generating highly reactive, partially reduced oxygen species, such as superoxides and hydrogen peroxide [49,50]. Thiols may also cause lipid peroxidation, producing hydroxyl radicals and cleaving proteins via a reaction that requires iron [56,57]. In the absence of metal ions, thiols protect against oxidative stresses.

Jiang et al. [58] indicated that a shift in the extracellular redox state in vivo could play an important role in RPE cell function and contribute to apoptosis of RPE cells undergoing oxidative stress. Because of this, the extracellular thiol/disulfide redox state may represent a physiologic environment in which other oxidative challenges, such as infection, activation of the immune system, or other stress conditions, result in enhanced RPE cell loss and the development of ARMD. Consequently, manipulation of the plasma thiol-disulfide redox state to a more reduced level may decrease the sensitivity of the retina and RPE cells to apoptosis from oxidative stress. Therefore, regulating the oxidation state of sulfur-containing amino acids may be an important strategy for controlling cellular damage and atherosclerotic lesions [59]. Although it needs further study, antioxidant therapy may inhibit vascular disease in patients who suffer from hyperhomocysteinemia and E-ARMD.

Our study had some limitations. We evaluated only a small number of subjects; thus, these findings may not be generalized to represent the entire E-ARMD patients’ population. We found that the OX-LDL level was associated with the plasma Hcy concentration. However, further work is required in large, case-control studies to determine the general applicability of our results, because of the multifactorial nature of E-ARMD disease.

### Conclusions

In conclusion, this study showed that the extracellular thiol state may represent a physiologic environment in which other oxidative challenges, such as hyperhomocysteinemia or other stress conditions related to macula condition, result in enhanced RPE cell loss and the development of ARMD. Change in the plasma thiol/disulfide redox state to a more reduced level may decrease the sensitivity of the retina and RPE cells to apoptosis from oxidative stress. Thiol and glutathione levels in the plasma of patients at risk for E-ARMD or with early ARMD may be therapeutically elevated to strengthen the antioxidative capacity of the retina and thus reduce the redox state of the retina.
